# 

**Published:** 1906-04

**Authors:** 


Souvenir of Swearingen.
Status of the Colored Brother.

VOL. XXI. APRIL, 1906. No. 10.

THE "RED BACK"

### TEXAS Medical Journal.

TWENTY-FIRST YEAR.

SUBSCRIPTION, $1.00 A YEAR, IN ADVANCE.

SINGLE COPIES, 10 CENTS.

PUBLISHED AT AUSTIN, TEXAS, BY

F. E. DANIEL, M. D.,

PROPRIETOR.

PRINTED BY THE VON BOECKMANN-JONES CO.

Entered at the Postoffice at Austin, Texas, as Second-class Mail Matter.

